# Supplementary material for: Survey from 61,748 schools in four States of India on sale of tobacco products near schools
Source: PLoS One. 2024 Apr 16;19(4):e0301893. doi: 10.1371/journal.pone.0301893 (PMC11020857; doi:10.1371/journal.pone.0301893)
Supplement: Attachment — Submitted filename: Response to Reviewers18thMarch2024.docx [file pone.0301893.s004.docx]

**RESPONSE TO QUERY** (31^st^ January 2024)

1. **Regression results indicate that the possibility of T-shops near primary schools is significantly higher than upper primary and secondary schools, which is against reality. It seems like either data cleaning is not done appropriately or the regression analysis is not conducted accurately.**

Thank you for the query. You are right, as the data suggests that in two states, the T-shops are higher in the upper primary and secondary schools as compared to primary, however, in pooled analysis, T-shops around primary schools are higher (Table 8).

2. **Similarly, the Chi-Square results show that more than 98 percent of shops are near co-education schools, whereas the regression results are not in line with these findings.**

According to Table 7, Chi-Square test indicates that there was a significant difference between the number of shops between co-education schools and other gender, however, the number of schools with tobacco shops in co-education schools (15790 / 60321) 26 percent while for girls (31.6 percent). Therefore, the results say the same in regression with girls and boys having higher odds ratio as compared to co-education (Table 8). Hope this resolves your query.

**RESPONSE TO QUERY** (28^th^ July 2023)

3. **As you have carried out a survey, there is an expectation that you as authors would have received formal permission/approval to collect, analyse and publish the data. Therefore, please provide confirmation from your institutional review board or research ethics committee (e.g., in the form of a letter or email correspondence) that ethics review was not necessary for this study. Please include a copy of the correspondence as an "Other" file."**

Thank you for your query. In terms of collection, analysis of data, it is part of the activities the schools are supposed to do as per the instructions (Orders) from the State Governments. As indicated earlier, this program implementation does not require ethics approval, so we do not have one at the moment. However, if this is a crucial requirement to proceed, we can apply for it and add it to the study. This process will take atleast two months or so. Please do let us know.

**RESPONSE TO QUERY** (25^th^ July 2023)

1. **Even if the data collected were not medical in nature, there would still be an expectation that you would have received some form of approval (or permission) to carry out the survey. Therefore please could you clarify whether you received specific permission to undertake the survey and provide the name of the body who provided this permission?**

As mentioned previously, each State Government gave Orders to the Education Department officials, Principals to implement this program and providing this information is one of the activities of that program and it is specifically mentioned in the Orders. A copy of the Order of one of the states has been uploaded in ‘Other’ *(please see Activity No.9 in the guidelines in the Appendix of the Government Order)*.

**RESPONSE TO QUERY** (7^th^ July 2023)

**1. Thank you for your response to the query and for clarifying that the survey was conducted by yourselves. Although the data collected were not medical in nature, please could you indicate whether there was any ethical oversight/permission/approval given to carry out the study (perhaps from the State Government or similar).**

Thank you for the query. As you rightly stated, the data was not medical in nature. Hence, there was no ethical approval/permission and consent required. However, as mentioned earlier, instructions/Government Orders were issued by the State Governments to the schools in these states to implement the program and update it on our web-based App.

*(a previous study's reference:* [*https://www.ncbi.nlm.nih.gov/pmc/articles/PMC7552856/*](https://www.ncbi.nlm.nih.gov/pmc/articles/PMC7552856/)*)*

**RESPONSE TO QUERY** (4^th^ July 2023)

**1. Thank you for providing further details about the survey. From your response it appears that the survey was not conducted by yourselves, but rather undertaken by the Ministry of Health & Family Welfare and then the data appears in the Unified District Information System for Education. You have accessed that data from the UDIS to undertake this study.

If this is the case, please could you provide details of how you accessed the data from the UDISE (i.e. is it publicly available and provided in an anonymised form). If not publicly available, please outline the permission required to access in your manuscript. This is not clear in your data availability statement.**

1R: Thank you for the query. This survey was conducted by us. It was a component of the Tobacco Free Educational Institution (ToFEI) program being run by us using our App in these four states. The guidelines for ToFEI were issued by the Ministry of Health & Family Welfare in 2019. Implementation of the program is being done by various agencies and in these four states it is being run by us for the State Governments.

We downloaded the initial list of schools and data from the UDISE that is available in public domain (<https://schooleducation.kar.nic.in/html/UDISECode.html>). This has been edited the *‘Materials and Methods’* section in the manuscript to clarify this.

**RESPONSES TO QUERIES** (received March 29, 2023):

1. **Please ensure that your manuscript meets PLOS ONE's style requirements, including those for file naming. The PLOS ONE style templates can be found at**

<https://journals.plos.org/plosone/s/file?id=wjVg/PLOSOne_formatting_sample_main_body.pdf> and

<https://journals.plos.org/plosone/s/file?id=ba62/PLOSOne_formatting_sample_title_authors_affiliations.pdf>

1R. Thank you. The changes have been done as per the PLOS ONE’s style requirements.

**2. You indicated that ethical approval was not necessary for your study. We understand that the framework for ethical oversight requirements for studies of this type may differ depending on the setting and we would appreciate some further clarification regarding your research. Could you please provide further details on why your study is exempt from the need for approval and confirmation from your institutional review board or research ethics committee (e.g., in the form of a letter or email correspondence) that ethics review was not necessary for this study? Please include a copy of the correspondence as an "Other"" file.**

2R: Thank you for the comment. This survey is a component of the Tobacco Free Educational Institutions being run by us for the State Governments. Government Orders to this effect were issued in these Indian states under which every school has to complete anti-tobacco activities. This survey is one of the activities of the program. The respondents were Principals/Teachers who reported the activity from schools on our App fulfilling the requirement of the Government Orders. Hence, this study did not need ethics committee approval *[Line 112 to 116 on pg4].*

**3. Please provide additional details regarding participant consent. In the ethics statement in the Methods and online submission information, please ensure that you have specified what type you obtained (for instance, written or verbal, and if verbal, how it was documented and witnessed). If your study included minors, state whether you obtained consent from parents or guardians. If the need for consent was waived by the ethics committee, please include this information.**

3R: Thank you for the comment. In this study, Orders were issued by the State Governments to report the activity on our App. The respondents were Teachers/Principals from schools who completed this activity fulfilling the requirement of the Government Orders. Hence, this study did not need participant consent and nor our study included any minors *[Line 112 to 116 on pg4].*

**4. In your Data Availability statement, you have not specified where the minimal data set underlying the results described in your manuscript can be found. PLOS defines a study's minimal data set as the underlying data used to reach the conclusions drawn in the manuscript and any additional data required to replicate the reported study findings in their entirety. All PLOS journals require that the minimal data set be made fully available. For more information about our data policy, please see http://journals.plos.org/plosone/s/data-availability #Ioc-recommended-repositories.**

**Upon resubmitting your revised manuscript, please upload your study’s minimal underlying data set as either Supporting Information files or to a stable, public repository and include the relevant URLs, DOIs, or accession numbers within your revised cover letter. For a list of acceptable repositories, please see http://journals.plos.org/plosone/s/data-availability#. Any potentially identifying patient information must be fully anonymized.**

**Important: If there are ethical or legal restrictions to sharing your data publicly, please explain these restrictions in detail. Please see our guidelines for more information on what we consider unacceptable restrictions to publicly sharing data:** [**http://journals.plos.org/plosone/s/data-availability#loc-unacceptable-data-access-restrictions**](http://journals.plos.org/plosone/s/data-availability#loc-unacceptable-data-access-restrictions)**. Note that it is not acceptable for the authors to be the sole named individuals responsible for ensuring data access.**

**We will update your Data Availability statement to reflect the information you provide in your cover letter.**

4R: Thank you for the comment. Data has been uploaded as Supporting Information file.

**5.  We note that you have stated that you will provide repository information for your data at acceptance. Should your manuscript be accepted for publication, we will hold it until you provide the relevant accession numbers or DOIs necessary to access your data. If you wish to make changes to your Data Availability statement, please describe these changes in your cover letter and we will update your Data Availability statement to reflect the information you provide**

Available data has been uploaded.

**6. We note that Figure 1 in your submission contain [map/satellite] images which may be copyrighted. All PLOS content is published under the Creative Commons Attribution License (CC BY 4.0), which means that the manuscript, images, and Supporting Information files will be freely available online, and any third party is permitted to access, download, copy, distribute, and use these materials in any way, even commercially, with proper attribution. For these reasons, we cannot publish previously copyrighted maps or satellite images created using proprietary data, such as Google software (Google Maps, Street View, and Earth). For more information, see our copyright guidelines:** [**http://journals.plos.org/plosone/s/licenses-and-copyright**](http://journals.plos.org/plosone/s/licenses-and-copyright)**. We require you to either (1) present written permission from the copyright holder to publish these figures specifically under the CC BY 4.0 license, or (2) remove the figures from your submission:**

6R: Thank you for the suggestion. We have removed this figure as this can be explained in the content.

**Comments to the Author**

**1. Is the manuscript technically sound, and do the data support the conclusions?

The manuscript must describe a technically sound piece of scientific research with data that supports the conclusions. Experiments must have been conducted rigorously, with appropriate controls, replication, and sample sizes. The conclusions must be drawn appropriately based on the data presented.**

**Reviewer #1: Yes**

**Reviewer #2: No**

**2. Has the statistical analysis been performed appropriately and rigorously?**

**Reviewer #1: Yes**

**Reviewer #2: No**

3**. Have the authors made all data underlying the findings in their manuscript fully available?

The** [**PLOS Data policy**](http://www.plosone.org/static/policies.action#sharing) **requires authors to make all data underlying the findings described in their manuscript fully available without restriction, with rare exception (please refer to the Data Availability Statement in the manuscript PDF file). The data should be provided as part of the manuscript or its supporting information, or deposited to a public repository. For example, in addition to summary statistics, the data points behind means, medians and variance measures should be available. If there are restrictions on publicly sharing data—e.g. participant privacy or use of data from a third party—those must be specified.**

**Reviewer #1: Yes**

**Reviewer #2: No**

**4. Is the manuscript presented in an intelligible fashion and written in standard English?

PLOS ONE does not copyedit accepted manuscripts, so the language in submitted articles must be clear, correct, and unambiguous. Any typographical or grammatical errors should be corrected at revision, so please note any specific errors here.**

**Reviewer #1: Yes**

**Reviewer #2: No**

5. Review Comments to the Author

Please use the space provided to explain your answers to the questions above. You may also include additional comments for the author, including concerns about dual publication, research ethics, or publication ethics. (Please upload your review as an attachment if it exceeds 20,000 characters)

Reviewer #1: Well conducted and described survey. Manuscript provides detailed statistical analysis. The recommendations provided could be a great asset for the enforcement agencies and policy makers. Further, this kind of surveys could be conducted in the other parts of country where, prevalence of tobacco consumption is high in adolescent age group.

**Reviewer #2: 1. Introduction section does not highlight the gap in literature and the contribution of the study. It seems like that the data collection is the only contribution. Reader is unable to identify the objectives and research question of the study.**

R2#1: Thank you for the points. Literature has been added in Line 86 to 90 on pg 3. The objectives are mentioned in Line 94 to 100 on pg 4. To our knowledge such a large and significant survey and its conclusions provides evidence for judicial action and to enforcement agencies and policy makers.

**2. Size variable consider less than 30 students in a school- is there exists any school in India with student population of less than 30?**

R2#2: Thank you for the query. Indeed, in a country with such a large population, there are many schools where the student enrolment is less than 30. As per the Unified District Information System for Education, in the state of Meghalaya, 4,627 (31% of total) schools have less than 30 students. In Karnataka 17,225 (24% of total) schools have less than 30 students.

**3. P#6, authors state, “Schools from all 133 districts and 1,763 Blocks (out of 1868) of the four States reported activity on the App.” If this true, then there is no district under consideration for the comparison group?**

3R: Thank you for the comment. This is a survey of the current status. Samples from 1763 blocks out of 1868 indicates that we have sampled schools from 94% of the administrative areas in these States. We aim to find out the percentage of schools where COTPA section 6(b) is being violated. Secondly, also to find out whether there was an association between the schools with shops selling tobacco within 100 yards and variables such as size, category, gender, location of schools. Finally, we also wanted to observe whether there is any relationship between the overall prevalence of tobacco use in the state and percentage of schools with Section 6(b) violation. Therefore, comparison group may not be needed.

**4. P#6, “On logistic regression modelling, primary school category had significant higher T-shops as compared to secondary (OR: 0.835) and higher secondary (0.829)”. These results are counter intuitive and counter factual as well.**

4R: Thanks for the correction. We have modified statement as “On logistic regression modelling, upper primary school category had significant higher T-shops as compared to primary only (OR: 1.15) while lower number T-shops were present in only primary schools as compared to secondary(OR.0.835) and higher secondary (0.829) (Table 5).” *[Line 184 to 186 on page 7]*

**5. On balance, the study concludes that girls only and boys only schools have higher chances of having T-shops near the schools. These results are interpreted mechanically without relating to theory and hence the authors are unable to provide any rationale to support the findings of the study.**

5R: Thank you for your suggestion. As per our findings, there were more number of tobacco shops near ‘girls only’ school as compared to CO-ED (co-education), however, the number of schools found in this category were not comparable thus findings only pave a way for further research. We have also modified the manuscript in the discussion section for better understanding *[Line 331 to 333 on pg15]*.

**6. The study relies on the reported results and does not providing any mechanism regarding the reporting biases- both type-I & II errors.**

6R: Thank you for your comment. We have mentioned about the potential reporting bias in the limitation of the study. I suppose reviewer meant TYPE I (ALSO KNOWN AS ‘α’) AND TYPE II (ALSO KNOWN AS ‘β’) ERRORS which are false positive and false negative. False-positive and false-negative results can also occur because of bias (observer, instrument, recall, etc.) Although type I and type II errors can never be avoided entirely, however, we have tried to reduce its likelihood by increasing the sample size (Banerjee A, Chitnis UB, Jadhav SL, Bhawalkar JS, Chaudhury S. Hypothesis testing, type I and type II errors. Ind Psychiatry J. 2009 Jul;18(2):127-31. doi: 10.4103/0972-6748.62274. PMID: 21180491; PMCID: PMC2996198.). This study has one of the highest sample size of number schools (61,748**)** reported till date.

**7. Results are poorly presented- screenshots of the table are given in the appendices.**

7R: As per the PlosOne requirements, tables have been added to the text.

6. PLOS authors have the option to publish the peer review history of their article ([what does this mean?](https://journals.plos.org/plosone/s/editorial-and-peer-review-process#loc-peer-review-history)). If published, this will include your full peer review and any attached files.


If you choose “no”, your identity will remain anonymous but your review may still be made public.


**Do you want your identity to be public for this peer review?** For information about this choice, including consent withdrawal, please see our [Privacy Policy](https://www.plos.org/privacy-policy).

Reviewer #1: No

Reviewer #2: No
